# Picolinic acid, a tryptophan metabolite, exhibits anabolic effects in muscle cells and improves lifespan and movement in *C. elegans*

**DOI:** 10.1093/gerona/glaf239

**Published:** 2025-11-04

**Authors:** Daniel Rivas, Ivan Baltasar-Fernandez, Abdelrahman AlOkda, Ahmed Al Saedi, David Karasik, Jeremy M Van Raamsdonk, Gustavo Duque

**Affiliations:** Bone, Muscle & Geroscience Group, Research Institute of the McGill University Health Centre, Montreal, Quebec, Canada; Bone, Muscle & Geroscience Group, Research Institute of the McGill University Health Centre, Montreal, Quebec, Canada; GENUD Toledo Research Group, Faculty of Sport Sciences, University of Castilla-La Mancha, Toledo, Spain; Bone, Muscle & Geroscience Group, Research Institute of the McGill University Health Centre, Montreal, Quebec, Canada; Division of Endocrinology, Boston Children’s Hospital, Boston, Massachusetts, United States; Department of Pediatrics, Harvard Medical School, Boston, Massachusetts, United States; The Musculoskeletal Genetics Laboratory, The Azrieli Faculty of Medicine, Bar-Ilan University, Safed, Israel; Bone, Muscle & Geroscience Group, Research Institute of the McGill University Health Centre, Montreal, Quebec, Canada; Bone, Muscle & Geroscience Group, Research Institute of the McGill University Health Centre, Montreal, Quebec, Canada; Dr. Joseph Kaufmann Chair in Geriatric Medicine, Department of Medicine, McGill University, Montreal, Quebec, Canada; (Biological Sciences Section)

**Keywords:** Aging, C. Elegans, Gerotherapeutics, Healthspan, Kynurenine, Tryptophan

## Abstract

Compounds promoting anabolic effects on muscle and bone may offer an ideal treatment for osteosarcopenia while potentially impacting healthspan and lifespan. We previously demonstrated the anabolic effects of picolinic acid (PIC), a tryptophan metabolite, on bone both *in vitro* and *in vivo*. However, its effects on muscle and potential additional effects on lifespan and healthspan are not yet fully understood. This study aimed to investigate PIC's effects on muscle cells *in vitro* and its impact on mobility and lifespan in an animal model. Murine C2C12 and human myoblasts were treated with PIC (1, 50, and 100 µM) or vehicle for 5 days. Myogenic regulatory factors (MRFs) were evaluated, and the fusion index and myotubules’ length were calculated at timed intervals (day 1, 3, and 5). *In vivo*, *Caenorhabditis elegans* were treated with increasing doses of PIC, and their lifespan and rate of movement (thrashing) were evaluated at timed intervals. PIC-treated myoblasts showed a higher and earlier expression of MRFs. On day 3, PIC-treated myotubes were significantly more fused and longer when treated with PIC than vehicle-treated controls. *C*. *elegans* treated with 1 mM of PIC showed a significantly longer lifespan. In addition, the mobility of PIC-treated *C. elegans* was significantly increased at all timed points. In conclusion, this study demonstrates that, besides its anabolic effect on bone, PIC has an anabolic effect on muscle, which is also associated with a longer lifespan in PIC-treated *C*. *elegans*. This evidence opens up promising avenues for further exploration of PIC as a novel therapy for osteosarcopenia with additional effects on healthspan and lifespan.

## Introduction

Aging causes muscle and bone loss through a mix of biological, hormonal, and lifestyle factors. The related conditions, known as sarcopenia (muscle loss) and osteoporosis (bone loss), are multifaceted and can greatly affect mobility, strength, and overall health. Osteosarcopenia, which is the concurrent presence of osteoporosis and sarcopenia, becomes more common with age [women: 14.3% (60-64 years) to 59.4% (≥75 years); men: 20.3% (60-64 years) to 48.3% (≥75 years)] and is more frequent in women.^1^ Osteosarcopenia significantly raises the risk of falls and fractures because of increased bone fragility, loss of muscle mass and strength, and poor balance.^1^ Despite its high prevalence, there are no drugs currently approved that have combined therapeutic effects on both muscle and bone.

Gerotherapeutics, which focus on addressing the molecular and biological mechanisms (hallmarks) of aging, could help manage osteosarcopenia by targeting underlying mechanisms common to aging-related bone and muscle loss, such as stem cell exhaustion, inflammaging, dysbiotic microbiota, oxidative stress, and cellular senescence.^2^ The principle of gerotherapeutics (and the global geroscience approach) is that these compounds could treat several age-related conditions by targeting the hallmarks of aging while preventing disability and frailty and extending healthspan.^3^ Indeed, osteosarcopenia is an optimal target for a gerotherapeutic since it is not only the combination of two highly prevalent chronic conditions that predispose older persons to adverse outcomes (ie, depression, falls, fractures, disability and frailty) but also the consequence of alterations in multiple hallmarks of aging.^2^

Tryptophan (Trp) is an essential amino acid and a precursor to bioactive metabolites.^4^ Trp metabolism in the gastrointestinal tract follows three pathways: (i) conversion by gut microbiota; (ii) the kynurenine pathway via tryptophan 2,3-dioxygenase (TDO) in the liver and indoleamine 2,3-dioxygenase (IDO) in immune and epithelial cells; and (iii) serotonin production in enterochromaffin cells.^5^ The kynurenine pathway is responsible for over 95% of all Trp degradation in mammals. It is mainly active in the liver, where approximately 90% of Trp is degraded under normal physiological conditions, and where all the necessary enzymes and substrates for the synthesis of two important end metabolites—picolinic acid (PIC) and nicotinamide adenine dinucleotide (NAD+), a coenzyme vital for redox reactions central to energy metabolism and DNA repair—are present.^6-8^ The pathway also exists outside the liver, although its contribution to Trp degradation usually remains minimal (5%-10%).^4^ However, it becomes more significant under pathological conditions involving immune activation, such as inflammaging or dysbiosis in the gut microbiota.^9-11^ The extrahepatic pathway does not include all the enzymes of the primary pathway, which results in the production of harmful intermediates like kynurenine at the expense of beneficial metabolites such as PIC and NAD+ in cases of unhealthy aging or chronic disease states. This phenomenon has been associated with age-related conditions, including neurodegeneration, depression, osteoporosis, sarcopenia, osteosarcopenia, and frailty.^9-12^

We have reported that PIC has strong bone anabolic (bone-forming) effects both *in vitro* and *in vivo*.^13^^,^^14^ This anabolic effect includes the induction of osteogenesis in human bone marrow stromal cells (BMSC),^13^ an increase in bone mass and quality in oophorectomized (OVX) C57BL/6 mice, and stimulation of bone formation in Sham-operated controls.^14^ However, despite the observed biological link between muscle and bone in osteosarcopenia^15^ and the synchronic effect that bone anabolics may have on muscle mass and function, the effect of PIC on muscle cells remains unknown. Therefore, in this study, we hypothesize that PIC has an additional anabolic effect on muscle, promoting muscle cell differentiation and enhancing mobility while extending lifespan in the nematode *Caenorhabditis elegans* (*C. elegans*), a model system for aging research.

## Methods

### Cell culture and myogenic differentiation

Mouse myoblast C2C12 cells (American Type Culture Collection, VA, USA) and Human Skeletal Muscle Myoblasts (HSMM) (Zenbio, NC, USA) were cultured in high glucose Dulbecco’s modified Eagle growth medium (DMEM; Wisent, Canada) supplemented with 10% fetal bovine serum (FBS) and 1% penicillin-streptomycin. To induce differentiation, cells were switched to differentiation media consisting of high-glucose DMEM supplemented with 2% horse serum and 1% penicillin-streptomycin for 7 days. The differentiation media were changed every two days. For PIC treatment, cells were seeded at a density of 45 000 cells/cm^2^ in a 6-well plate and incubated at 37 °C under 5% CO_2_ for 24 h in growth media. The growth media were then replaced with differentiation media containing increasing concentrations of PIC (1, 50, 100, and 500 µM) or vehicle for 7 days.

### Cell viability assay (MTS assay)

To determine nontoxic concentrations, cells were treated with varying concentrations of PIC, and cell viability (MTS assay) was measured. Concentrations that allowed more than 80% of cells to survive at all time points were considered nontoxic and were used in further experiments. Murine C2C12 and human myoblasts were seeded at a density of 10 000 cells/well in a 96-well plate and incubated at 37 °C for 24 h in growth media. Cells were treated with increasing concentrations of PIC (1, 50, 100, and 500 µM) or vehicle. After 24 h, 48 h, and 120 h in culture, cell viability was evaluated using a 3-(4,5-dimethylthiazol-2-yl)-2,5-diphenyltetrazolium bromide (MTT) assay kit (Cat no. 11465007001; Roche Applied Science), according to the manufacturer’s protocol. After incubation, 10 µL of the MTT labeling reagent (final concentration 0.5 mg/mL) was added to each well. The microplate was incubated for 4 h in a humidified atmosphere at 37 °C, 5% CO_2._ Solubilization solution (100 µL) was added to each well. The plate was allowed to stand overnight in the humidified atmosphere of the incubator. Upon complete solubilization of purple formazan crystals, absorbance was measured at 550 nm using an Infinite M200 Pro Tecan Plate Reader (Tecan Austria GmbH, Austria). Cell viability was calculated using the following formula = (absorbance treated-absorbance blank)/(absorbance nontreated-absorbance blank) × 100%.

### Confocal microscopy

Murine C2C12 and human myoblasts were cultured at a density of 45 000 cells/cm^2^ and differentiated on µ-Slide 8 well ibiTreat chambers (80806, ibidi, Germany). Cells were treated with increasing doses of PIC or vehicle as previously described. To assess the myoblasts’ ability to fuse and form myotubes, cells were fixed at specific intervals (day 1, 3, and 5) in 4% (v/v) paraformaldehyde for 30 min, then, washed three times with cold PBS. Cells were permeabilized with 0.1% (v/v) Triton-X100 in PBS (PBST) for 5 min and stained with Phalloidin-iFluor 488 reagent (ab176753, Abcam, USA) for 90 min. After three additional washes with cold PBS, mounting media with DAPI was added to each well. Phalloidin labels actin filaments in muscle, enabling visualization of the myotubes and their DAPI-stained nuclei. Cells were imaged using a Zeiss LSM780 Laser Scanning Confocal Microscope. A myoblast containing three or more nuclei was classified as a myotube. Myotubes were evaluated based on their length and fusion index. The fusion index was calculated as the number of nuclei within a myotube divided by the total nuclei in a field of view. Fusion index is a key indicator of myogenic differentiation, essentially quantifying how effectively myoblasts are merging to create muscle fibers. A higher fusion index signifies greater differentiation and muscle formation capacity. Length measurements and fusion index quantification were performed using ImageJ software. All images were acquired under consistent fluorescent settings, only adjusted for focus, with 8-10 representative images captured per condition.

### Expression of myogenic regulating factors by Western blot analysis

Post-treatment myotubes were washed with PBS before being lysed with RIPA lysis buffer containing protease inhibitors and centrifuged at 17 000 *g*, 4 °C for 20 min to remove insoluble material. The supernatant was carefully collected (to avoid disturbing the pellet at the bottom) into another tube and stored at −80 °C till required. Protein concentrations were calculated using the Bio-Rad protein assay kit and aliquoted into the SDS electrophoresis sample buffer. Equal concentrations of protein lysates (10 µg per well) were loaded and electrophoresed on a Mini-PROTEAN TGX Stain-Free Gel (4568086, Bio-Rad) at 100 V from anywhere between 90 and 120 min before being electro-transferred to a polyvinylidene fluoride (PVDF) membrane overnight at 4 °C at 10 volts. The membranes were then blocked in 5% (w/v) skim milk or 5% (w/v) BSA in Tris-buffered saline, 0.1% Tween-20 (TBST) for 1 h, and then, incubated overnight at 4 °C under light agitation with primary antibodies against Myosin IIa (1:1000, Cell Signaling Technology, 3403S), myoblast determination protein 1 (MyoD) (1:1000, Cell Signaling Technology, 13812S), Myogenin (1:400, Abcam, ab1835), and GAPDH (1:2000, Cell Signaling Technology, 5174S). Following the overnight incubation, the membranes were thoroughly washed in TBST and incubated for 2 h under light agitation with the appropriate corresponding HRP-conjugated secondary antibody (Goat Anti-Rabbit, 170-6515 or Goat Anti-Mouse, 170-6516, Bio-Rad). The membrane was again thoroughly washed in TBST, developed using Clarity Western ECL Substrate and imaged using a Chemidoc MP Imaging system (Bio-Rad) to allow viewing and quantifying protein expression banding. Proteins were normalized against GAPDH.

### Lifespan assay

N2 (wildtype) *C. elegans* were bleached, and eggs were allowed to synchronize in M9 buffer, rotating for 20 h. The L1 larvae were transferred to NGM plates seeded with OP50 *Escherichia coli* and left for 48 h to reach young adulthood (YA). The YAs were transferred by picking to plates containing different concentrations of PIC (0.1, 1, 2, and 5 mM) and allowed to lay eggs overnight. The adults were removed, and the progeny were allowed to grow until the animals reached YA stage before transferring them to 50 µM FUdR plates with PIC. The survival of animals was checked every 2-4 days. Worms were transferred to fresh PIC-FUdR plates when needed.

### Thrashing

Once worms reached the appropriate age, a volume of 2 mL of M9 buffer was added to the worms, and they were left to acclimatize for 1 min before capturing 1 min video at 14 FPS using WormLab (MBF Bioscience). The videos were analyzed using wrMTrck plugin for the open-source image processing software, Fiji. Worm tracks were only considered for analysis when at least 420 frames (30 s) of movement were captured. The results were pooled from three biological replicates.

### Statistical analysis

Unless otherwise stated, all data are expressed as the mean ± standard deviation (SD) of a minimum of three replicate determinations. Mixed model repeated measures ANOVA was used to analyze the effects of PIC on C2C12 and human myoblast proliferation, length, fusion index and myogenic factors levels. All analyses were performed separately in C2C12 myoblasts and human myoblasts. The experiments were entered in the model as random effects while treatment (PIC 1, 50, and 100 μM, and vehicle) and assessment (day 1, 3, and 5) as fixed effects. Bonferroni’s *post hoc* tests for multiple comparisons were conducted to evaluate the effects of PIC treatment compared to the vehicle (between group comparisons) and across the different time points (within group comparisons). For lifespan experiments, GraphPad Prism 5 software was used to generate lifespan graphs and OASIS software (version 2) was used for statistical analysis to determine mean lifespan.^16^  *p* Values were calculated using the log-rank (Mantel–Cox) method and Bonferroni correction was performed to account for the multiple comparisons. Otherwise, quantitative data were analyzed using GraphPad Prism 5. A one-way ANOVA was performed with a Dunnett’s Multiple Comparisons Test. A value of *p* < .05 was used to establish statistical significance and *p*-values are presented in the figure legend.

## Results

### PIC has a cell-specific effect on proliferation in murine C2C12 and human myoblasts

Figure S1 (see online supplementary material for a color version of this figure) shows the effects of 1, 3, and 5 days of supplementation of increasing doses of PIC on estimates of viable cell numbers determined by the absorbance of the colored formazan product, which is directly proportional to the number of viable cells. In C2C12 myoblasts (Figure S1A, see online supplementary material for a color version of this figure), a similar number of viable cells was observed on day 1 in myoblasts treated with 1 µM (92.9 ± 3.8%; *p *= .557), 50 µM (92.4 ± 2.9%; *p *= .413), and 100 µM (92.4 ± 3.9%; *p *= .413) PIC compared to the vehicle (100.0 ± 4.2%). However, a lower number of viable cells was observed in murine myoblasts treated with 500 µM of PIC (78.2 ± 13.2%; *p *< .001). On day 3, a similar number of viable cells was observed in murine myoblasts treated with 1 µM PIC (91.0 ± 7.7%; *p *= .156) compared to the vehicle (100.0 ± 10.2%), whereas a lower number of viable cells was observed in murine myoblasts treated with 50 µM (86.5 ± 6.5%; *p *= .004), 100 µM (84.0 ± 6.0%; *p *< .001), and 500 µM (81.2 ± 9.5%; *p *< .001) of PIC. On day 5, a similar number of viable cells was observed in murine myoblasts treated with 1 µM (95.6 ± 4.8%; *p *= .999), 50 µM (92.3 ± 8.1%; *p *= .386), and 100 µM (90.1 ± 10.0%; *p *= .083) of PIC compared to the vehicle (100.0 ± 4.6%), whereas a lower number of viable cells was observed in myoblasts treated with 500 µM of PIC (84.2 ± 12.8%; *p *< .001). In human myoblasts (Figure S1B, see online supplementary material for a color version of this figure), a similar number of viable cells was observed in myoblasts treated with 1, 50, and 100 µM PIC compared to the vehicle on day 1, 3, and 5 (all comparisons *p *> .05). After performing the viability assays and excluding the 500 µM dose due to its high toxicity, the concentrations of 1, 50, and 100 µM were selected for further experiments.

### Treatment with PIC increases fusion index in murine and human myoblasts

**Figure 1.**
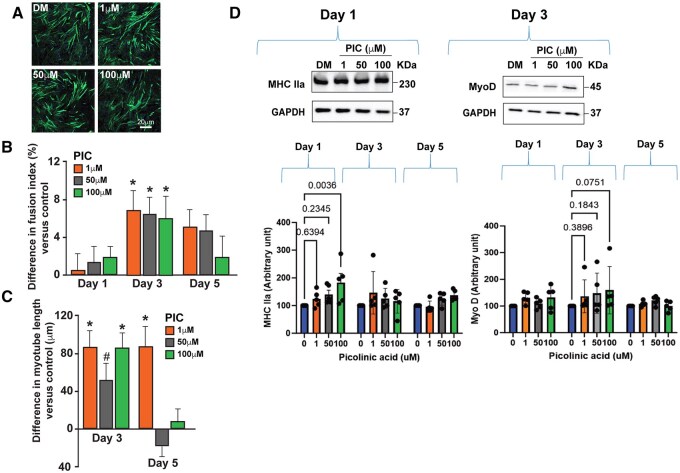
*In vitro* data showing the effect of picolinic acid (PIC) on C2C12 murine differentiation. (A) Representative confocal images showing actin filaments of differentiated murine myotubes on day 3 of differentiation. (B) PIC showed a significantly increased fusion index on day 3 at all tested PIC concentrations (*p* < .01). (C) PIC significantly increased myotube length on day 3 at 1 and 100 µM PIC concentration and at 1 µM at day 5 of differentiation. (D) The effect seen on myoblast fusion index and length was associated with increased expression of myogenic factor Myosin IIa at 100 µM PIC concentration on day 1 only (*p* = .002), but not with an increased expression of MyoD on any day (all *p* > .05). ^*^Indicates significant differences compared to vehicle (*p* < .05).

**Figure 2.**
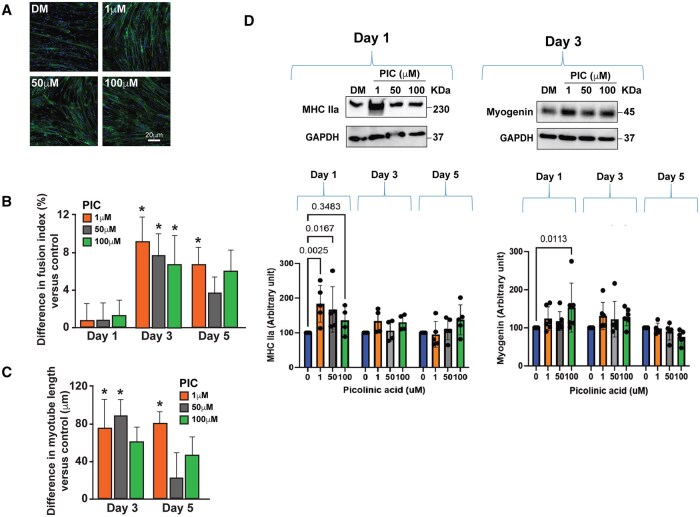
*In vitro* data showing the effect of picolinic acid (PIC) on human skeletal muscle myoblast (HSMM) differentiation. (A) Representative confocal images showing actin filaments of differentiated human myotubes on day 3 of differentiation. (B) PIC showed a significantly increased fusion index on day 3 at all tested PIC concentrations (*p* < .01) and on day 5 at lower concentrations (*p* < .005). (C) PIC significantly increased myotube length on day 3 at 1 and 50 µM PIC concentration and at 1 µM on day 5 of differentiation. (D) The effect seen on myoblast fusion index and length was associated with increased expression of Myosin Heavy Chain IIa (MHC IIa)at 1 and 50 µM PIC concentrations on day 1 only (both *p *< .05), and with an increased expression of Myogenin at 100 µM PIC concentrations on day 1 only (*p *= .009). ^*^Indicates significant differences compared to vehicle (*p *< .05).

Confluent (90%) cultures of murine and human myoblasts were differentiated into myotubes and treated with PIC (1, 50, and 100 µM) or vehicle for 5 days. To examine whether PIC stimulated myotube formation, we measured their fusion index using confocal microscopy on day 1, 3, and 5 of differentiation. Figures 1 and 2 show micrographs of control myotubes and PIC-treated cells taken on day 1, 3, and 5 of treatment (Figures 1A and B for murine and 2A and B for human cells). At day 1, the fusion index in both murine and human cells was comparable across PIC doses of 1, 50, and 100 µM to that of the vehicle (all *p *> .05). By day 3, murine cells exhibited a higher fusion index than vehicle when treated with PIC at concentrations of 1 (Δ = 7.4%; *p *= .001), 50 (Δ = 6.6%; *p *= .003), and 100 µM (Δ = 6.5%; *p *= .004). At day 5, murine cells showed a trend toward an increased fusion index with 1 (Δ = 4.9%; *p *= .060) and 50 µM (Δ = 4.8%; *p *= .075), but not with 100 µM (Δ = 2.8%; *p *= .869).

In human cells, at day 3, a higher fusion index was observed when treated with PIC at 1 µM (Δ = 9.3%; *p *= .001), 50 µM (Δ = 7.4%; *p *= .009), and 100 µM (Δ = 7.7%; *p *= .007) concentrations. By day 5, only treatment with 1 µM PIC resulted in a higher fusion index than vehicle (Δ = 6.7%; *p *= .033), whereas this effect was not noted at 50 (Δ = 3.5%; *p *= .889) or 100 µM PIC (Δ = 5.6%; *p *= .114).

### Treatment with PIC increases myotube length

We measured myotube length on day 3 and 5 of the differentiation to examine whether PIC stimulated myotube growth. Figures 1C and 2C show micrographs of control myotubes and PIC-treated murine (Figure 1C) and human (Figure 2C) cells taken on day 3 and 5 of treatment. At day 3, longer myotubes were observed in murine cells treated with 1 (Δ = 85.2 µm; *p *= .001) and 100 µM PIC (Δ = 83.9 µm; *p *= .001) compared to vehicle, whereas no significant differences were noted in cells treated with 50 µM PIC (Δ = 53.6 µm; *p *= .060). By day 5, murine cells treated with 1 µM PIC exhibited longer myotubes than vehicle (Δ = 88.2 µm; *p *< .001), while no significant differences in myotube length were observed in cells treated with 50 (Δ = −15.1 µm; *p *= .999) and 100 µM PIC (Δ = 4.7 µm; *p *= .999).

In human cells, at day 3, treatment with 1 (Δ = 75.7 µm; *p *= .044) and 50 µM PIC (Δ = 89.2 µm; *p *= .010) resulted in longer myotubes compared to vehicle, whereas no significant differences were noted in cells treated with 100 µM (Δ = 66.9 µm; *p *= .095). By day 5, only human cells treated with 1 µM PIC showed significantly longer myotubes than vehicle (Δ = 86.0 µm; *p *= .014), whereas no significant differences were found between vehicle and the 50 (Δ = 25.3 µm; *p *= .999) and 100 µM PIC treatments (Δ = 55.0 µm; *p *= .295).

### PIC increases myogenic factor levels in differentiating murine and human myoblasts

Figure 1D shows the expression of MHC IIa and MyoD in C2C12 myoblasts treated with different doses of PIC and the vehicle. Murine myoblast treated with 1 and 50 µM PIC in the differentiation media did not exhibit higher levels of expression of the myogenic factor MHC IIa on day 1 compared to vehicle (both *p *> .05). However, those treated with 100 µM PIC ­demonstrated higher expression of MHC IIa at day 1 than vehicle (Δ = 83.0 a.u.; *p *= .002). By day 3 and 5, MHC IIa expression levels were similar across the 1, 50, and 100 µM PIC-treated groups compared to the vehicle (all *p *> .05). Regarding MyoD expression, no significant differences were observed between murine myoblasts treated with PIC compared to the vehicle at any of the evaluated concentrations and time points (all *p *> .05).

Figure 2D shows the expression of MHC IIa and Myogenin in human myoblasts treated with different doses of PIC and the vehicle. On day 1, only treatment with PIC at 1 (Δ = 83.5 a.u.; *p *= .001) and 50 µM (Δ = 67.3 a.u.; *p *= .011) resulted in higher levels of MHC IIa expression compared to vehicle, while similar expression of MHC IIa was observed between myoblasts treated with 100 µM and the vehicle (Δ = 35.8 a.u.; *p *= .644). By day 3 and 5, MHC IIa expression was comparable across PIC treatments relative to the vehicle (all *p *> .05). Regarding Myogenin, human myoblasts treated with 1 and 50 µM PIC exhibited similar Myogenin expression compared to the vehicle on day 1, 3, and 5 (all *p *> .05). However, myoblasts treated with 100 µM PIC showed higher Myogenin expression than the vehicle on day 1 (Δ = 53.2 a.u.; *p *= .009), with expression levels similar to those of the vehicle on day 3 and 5 (all *p *> .05).

### PIC prolongs lifespan in *C. elegans*

Having demonstrated a beneficial effect of PIC on muscle cells in culture, we next examined the effects of PIC in a whole organism. For this, we chose to use the worm *C. elegans* where we could test the effect on body wall muscle function using a thrashing assay and measure lifespan. Before conducting lifespan experiments, we assessed the potential toxicity of PIC at doses ranging from 0.1 mM to 20 mM by observing development time. We found that development at 20 mM PIC was markedly slower than control, while concentrations of 5 mM and less had little or no effect on development time (not quantified).

**Figure 3.**
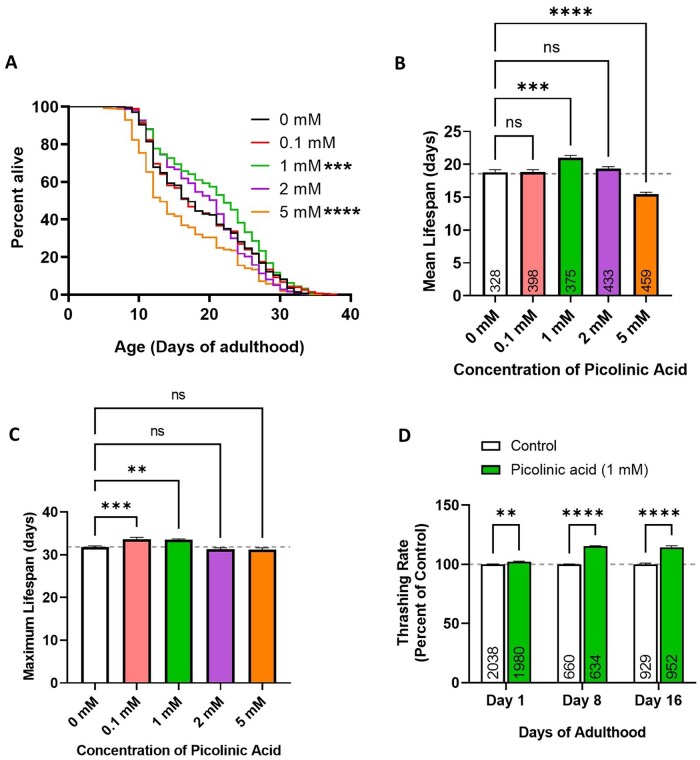
Picolinic acid (PIC) increases lifespan and motility in *C. elegans.* (A, B) *C. elegans* worms were treated with concentrations of picolinic acid ranging from 0.1 mM to 5 mM. 1 mM PIC significantly increased worm lifespan, while 5 mM PIC decreased lifespan. (C) Maximum lifespan was calculated as the average of the 20 longest-lived worms in each group. Maximum lifespan was significantly increased in worms treated with 0.1 mM and 1 mM PIC. (D) The effect of PIC on worm motility was determined by measuring the thrashing rate in liquid. Treatment with the lifespan-extending dose of 1 mM PIC resulted in increased movement in aging worms at day 8 and 16 of adulthood. Three biological replicates were performed. Statistical significance was determined using the log-rank test in panel A, a one-way ANOVA with Dunnet’s multiple comparisons test in panels B and C, and a two-way ANOVA with Šidák’s multiple comparisons test in panel D. Error bars indicate standard error of the mean. ^**^p < .01, ^***^p < .001, ^****^p < .0001.

To address whether PIC has a positive effect on the lifespan of *C*. *elegans*, wild-type N2 worms were treated with increasing concentrations of PIC (0.1, 1, 2, and 5 mM). The results showed that compared with the control group (equal volume sterile water was used as control), treatment with 1 mM PIC significantly increased the lifespan of the worms, with the mean lifespan increasing from 18.8 days in control to 21.0 days in the PIC-treated worms (Figure 3A and B). Maximum lifespan was also significantly increased by treatment with 1 mM PIC (Figure 3C and Table S1).

### PIC improves mobility in *C. elegans*

Mobility is a commonly used indicator of health and an indirect marker of muscle function. As the lifespan in worms exposed to 1 mM PIC was increased, we wished to explore the positive effect on health by exposure to PIC with the observation of mobility. To do this, the number of head-to-tail body bends in liquid (thrashing rate) was assessed in wild-type worms (strain, N2) on day 1, 8, and 16. Treatment with 1 mM PIC significantly increased the rate of thrashing in aged worms at both day 8 and day 16 of adulthood by approximately 15% (Figure 3D).

## Discussion

In this study, we demonstrated that, as shown in bone,^13^^,^^14^ PIC has an anabolic effect on myoblasts at the same doses previously tested in BMSC obtained from mice and humans.^13^^,^^14^ Interestingly, PIC also improved mobility and lifespan in *C. elegans*.

The kynurenine pathway of Trp metabolism has generated increasing interest, especially in the context of the regulatory effects that several of its metabolites have on the hallmarks of aging and their role in the development of multiple age-related diseases.^5-12^ Under normal conditions, 90% of Trp is metabolized in the liver to produce NAD+ and PIC, which are bioactive compounds that play roles in various biological processes.^6-8^ In addition to the anabolic effect of PIC on bone,^13^^,^^14^ both metabolites play a crucial role in supporting cellular antioxidant defenses and can indirectly contribute to reducing oxidative stress.^17-19^ NAD+ also participates in various biological functions such as proteostasis, mitochondrial activity, immune pathways, gene expression, and apoptosis,^20^ all of which are considered hallmarks of aging.^21^

In contrast, in cases of alterations associated with aging, such as dysbiotic microbiota and inflammaging, levels of toxic inflammatory metabolites such as kynurenine and kynurenic acid accumulate due to the absence of the required enzymes for kynurenine metabolism in extrahepatic tissues.^5^^,^^6^ These metabolites have a harmful effect on multiple organs and tissues (predominantly from mesenchymal origin) that include alterations in stem cell differentiation (exhaustion), inflammaging, fat accumulation in muscle and bone, dysfunctional autophagy, and apoptosis.^22^ Interestingly, these are the exact cell mechanisms involved in the pathogenesis of osteosarcopenia,^2^ allowing us to hypothesize that this condition is closely associated with alterations in the kynurenine pathway^10^ and that targeting this pathway could become a novel therapeutic approach with a dual beneficial effect on muscle and bone.

In this study, we examined the impact of PIC on two different cell models previously used to investigate the effect of therapeutic compounds on sarcopenia.^23^ We evaluated how PIC influences three myogenic regulatory factors (MRFs). MyoD is one of the earliest MRFs expressed and plays a crucial role in establishing the myogenic lineage. The other two assessed factors (MHC IIa and Myogenin) are expected to increase their expression at a more advanced stage of differentiation. MHC IIa is linked to muscle contraction, and Myogenin is vital for terminal muscle cell differentiation and muscle fiber formation. Both PIC-treated cell types exhibited an early (day 1) and significant increase in MHC IIa. However, their concentration-response patterns differed, with C2C12 cells showing a significant increase in MHC IIa only at 100 µM PIC, while human myoblasts responded to lower doses. Additionally, C2C12 cells showed a trend toward higher MyoD expression at day 3 with 100 µM PIC, with no observed changes in myogenin (data not shown). Conversely, PIC-treated (100 µM) human myoblasts displayed early elevated levels of Myogenin expression. Overall, these findings suggest that PIC’s molecular effects on differentiating myoblasts indicate a transient or partial, cell-specific activation of myogenic pathways. Such dissociation is common in differentiation studies and may arise from stress-related cellular responses that modify gene expression without necessarily affecting complete maturation.

Additionally, as observed in the transcription factors, PIC caused an early rise in the fusion index. The fusion index usually starts after 4-7 days of treatment with differentiation medium and continues through day 7-9. In our experiments, at day 3, PIC significantly increased the fusion index at all concentrations and in both cell lines, indicating that PIC could promote the formation of functional skeletal muscle fibers and confirming its pro-myogenic activity *in vitro*. Moreover, longer myotubes were observed at day 3 in PIC-treated C12C12 and human myoblasts, which also suggests successful fusion of multiple myoblasts into multinucleated myotubes. Overall, our results from transcription factors and the fusion index suggest that PIC has an anabolic effect on differentiating myoblasts. Of note, this anabolic effect was seen using the same PIC concentrations as those used by our group in BMSC.^13^^,^^14^

Additionally, we observed a beneficial effect of PIC on the mobility and lifespan of *C. elegans*. Higher mobility indicates better or maintained muscle function, especially of the body wall muscles responsible for movement. It suggests that PIC supports or improves muscle performance. Interestingly, PIC treatment also extended lifespan in this model; however, additional studies will be needed to determine the exact mechanisms by which PIC acts to extend longevity in *C. elegans*. In these studies, it will be important to include additional controls to test for the effect of PIC on pH and osmolality, and also to determine the extent to which PIC is acting directly on the worm versus indirect effects of PIC on the bacteria.

Indeed, our observed effects of PIC on *C. elegans* closely resemble those seen in other kynurenine metabolites with high potential to become gerotherapeutics. Knocking down genes involved in pro-aging elements of the kynurenine pathway has also been shown to extend lifespans in worms and flies.^24^^,^^25^ Additionally, using knockdown of *haao-1*, the fourth gene encoding the enzyme 3-hydroxyanthranilic acid (3HAA) dioxygenase (HAAO), Dang et al. extended lifespan by about 30% and delayed age-related health decline in *C. elegans.*^26^ This lifespan extension was mediated by increased physiological levels of the HAAO substrate 3HAA. Similar to PIC, 3HAA enhances oxidative stress resistance and activates the Nrf2/SKN-1 oxidative stress response. Moreover, the group demonstrated that female *Haao* knockout mice, as well as aging wild-type male mice fed a diet supplemented with 3HAA, were also long-lived. Whether PIC has a comparable effect on normally aged mice remains to be explored.

Substantial evidence suggests that various kynurenines may support bone metabolism, though their effects on muscle remain less defined. Elevated serum levels of 3-HAA, PIC, QUIN, and NAD+ have been associated with higher bone mineral density (BMD) and a lower risk of fractures in humans.^10^ Furthermore, the Hordaland Health Study (HUSK) identified positive correlations between xanthurenic acid, 3-HAA, and BMD in older adults.^27^ The role of kynurenic acid (KYNA) in bone health appears to be complex, with mixed findings. In aged C57BL/6 mice, high doses of KYNA were linked to a reduction in trabecular number and thickness, as observed through micro-CT analysis.^28^ Conversely, research involving younger mice of the same strain indicated that KYNA facilitates osteogenesis via the Wnt/β-catenin pathway.^29^ These findings suggest that the effects of KYNA on bone cells may depend on factors such as age and gender, which should be considered in future animal studies testing the effect of PIC on muscle.

Overall, although targeting the kynurenine pathway seems to be a promising approach to osteosarcopenia and other age-related conditions (ie, neurodegenerative, depression), there are challenges in targeting it for aging interventions. The pathway is complex and highly regulated, with metabolites that can have opposing effects depending on context, concentration, and tissue specificity. Balancing these metabolites to avoid unintended consequences, such as exacerbating neurotoxicity or immunosuppression, will be key in developing safe and effective gerotherapeutics. In fact, an advantage that PIC has over all the other kynurenine metabolites is that PIC is produced via a nonenzymatic conversion, which occurs when amino-ß-carboxymuconate-semialdehyde-decarboxylase (ACMSD; EC 4.1.1.45) is saturated with substrate allowing the production of quinolinic acid (QUIN). Under normal conditions, these two pathways control equal flux.^4^ Administration of exogenous PIC is not expected to alter this process, and thus, could constitute a safe therapeutic intervention, as demonstrated by the lack of toxicity observed in PIC-treated OVX mice and their sham-operated controls.^14^

In conclusion, while initial findings suggest that PIC may facilitate myogenesis *in vitro* (as was also shown for bone cells) and prolong lifespan and mobility in *C. elegans*, further experimental studies are needed to thoroughly assess the potential mechanism of action of PIC on muscle and its potential as a gerotherapeutic agent. Understanding its effects on bone and muscle in murine models and aging populations, as well as its mechanisms of action, will be essential for determining its suitability and efficacy in treating osteosarcopenia and promoting healthy aging.

## Supplementary Material

glaf239_Supplementary_Data

## Data Availability

Data available on request.
