# Supplementary figures and images for: Picolinic acid, a tryptophan metabolite, exhibits anabolic effects in muscle cells and improves lifespan and movement in C. elegans
Source: J Gerontol A Biol Sci Med Sci. 2025 Nov 4;81(1):glaf239. doi: 10.1093/gerona/glaf239 (PMC12758968; doi:10.1093/gerona/glaf239)

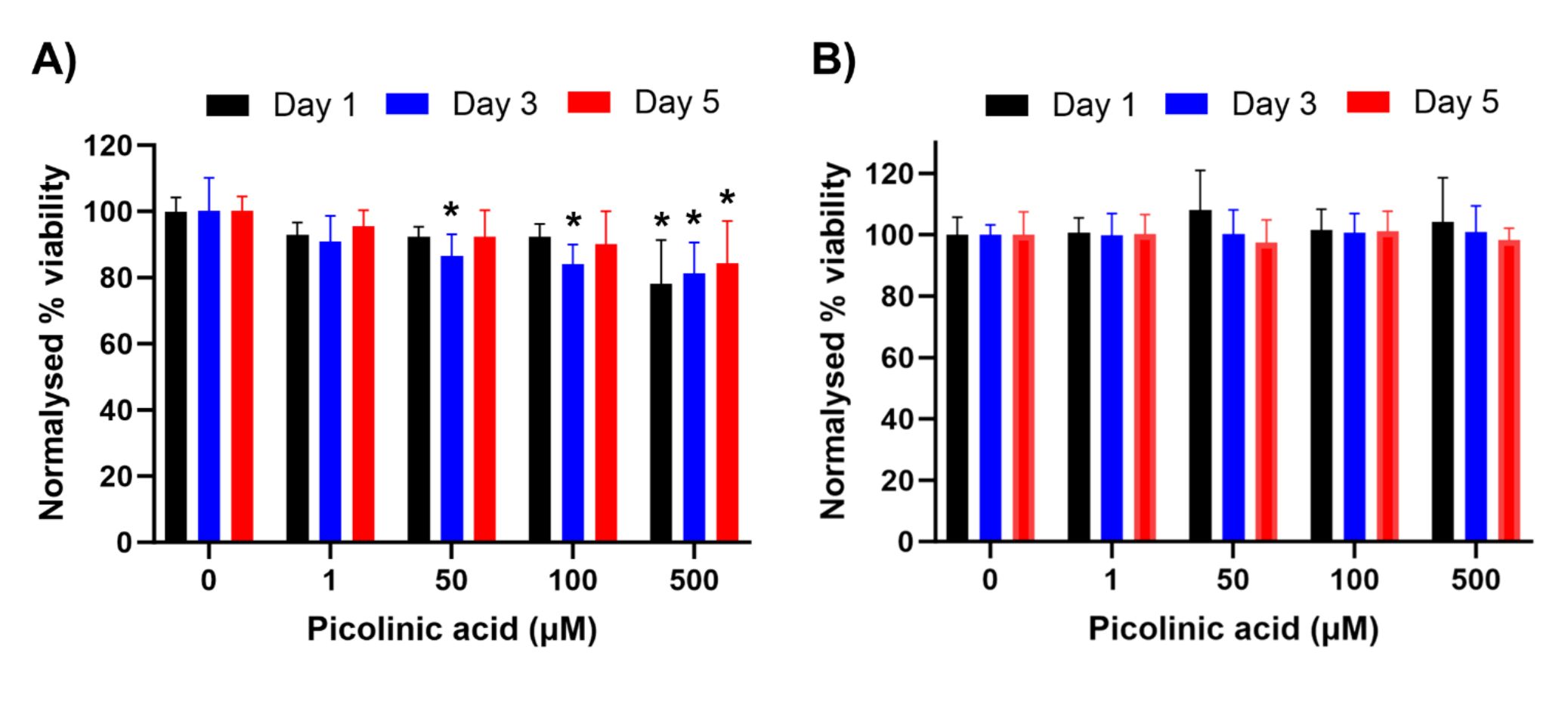

Supplement: glaf239_Supplementary_Data [file glaf239_supplementary_data.zip › Suppl Figure 1.tif]
